# Evaluation of Dental Professionals’ Knowledge and Attitude Regarding the Diagnosis of Oral Cancer Through Histopathological Examination of Granulation Tissue

**DOI:** 10.7759/cureus.44744

**Published:** 2023-09-05

**Authors:** Monika Kumari, Hina Naim Abdul, Chirag Vyas, Ambar Khan, Reya Shree, Garima Sharma

**Affiliations:** 1 Public Health Dentistry, Divya Jyoti College of Dental Sciences and Research, Ghaziabad, IND; 2 Prosthetic Dental Sciences, College of Dentistry, Jazan University, Jazan, SAU; 3 Public Health Dentistry, Indira Gandhi District Hospital Mandsaur, Mandsaur, IND; 4 Periodontology and Implantology, Divya Jyoti College of Dental Sciences and Research, Ghaziabad, IND; 5 Oral Medicine and Radiology, Teerthanker Mahaveer Dental College and Research Centre, Moradabad, IND

**Keywords:** oral cancer, granulation tissue, diagnosis, dentist, biopsy

## Abstract

Introduction: The current cancer trend in India has reported an alarming increase in cancer of the lip, throat, and oral cavity. Few dentists are aware that malignant neoplasms can also occur in the form of granulation tissue and periapical granulomas. However, most dentists agree that biopsies are essential to diagnose oral cancer. This makes the timely diagnosis of oral cancer dependent upon the histopathological examination of the granulation tissue by the dentist.

Aim: The main aim of this study was to evaluate the knowledge and attitudes of dentists regarding the diagnosis of oral cancer through histopathological analysis of granulation tissue.

Method: A cross-sectional study was conducted on dentists who were residents of Uttar Pradesh, India. Two hundred and fifty study participants enrolled to complete a self-structured questionnaire containing 15 closed-ended questions. The study was conducted over a period of three months, from June to August 2020. Descriptive statistics were analyzed using IBM SPSS Statistics for Windows, Version 21.0 (Released 2012; IBM Corp., Armonk, New York, United States, and the t-test was performed. A significance level of P ≤ 0.05 was used to determine the statistical significance of quantitative variables.

Results: The study findings revealed that only a small percentage of dentists (47.2%) were aware of the importance of biopsies in diagnosing oral cancer. Moreover, a minority (14.4%) had conducted biopsies and submitted samples for histopathological analysis. Of them, 10% were aware that in clinical practice, malignant tumors can also manifest as periapical granulomas, granulation tissue, gingivitis, and other conditions.

Conclusion: Dentists should proactively submit any granulation tissue for histopathological examination. Oral healthcare providers must maintain a high level of suspicion, develop a range of potential or differential diagnoses for oral cancer, and take necessary measures to attain a definitive diagnosis. This may include considering a referral to a specialist for the treatment of oral cancer.

## Introduction

Cancer of the oral cavity usually begins as a small, unrefamiliar, unexplained growth or ulceration that involves the lips, cheek, tongue, palate, base of the mouth, and the sinuses of the oral cavity. Oral cancer is the sixth most typical malignancy worldwide and a major public health issue that affects both industrialized and developing nations. India has the largest number of oral cancer cases and one-third of the total burden of oral cancer globally [1]. India reports roughly 77,000 new cases of oral cancer per year [2], which accounts for nearly 25% of all cases worldwide [3]. It is frequently characterized by aggressive behaviour and an unfavourable prognosis, but if detected early it is generally associated with a better disease. However, approximately 70% of these cases are not reported until they are in the advanced stages of cancer. This late-stage detection reduces the chances of recovery and survival rate of the patient [4]

Oral cancer is associated with various risk factors like periodontal illnesses, tobacco, alcohol, etc. Periodontal illness is also a high-risk consideration for oral malignancy, and it has a higher incidence among the Indian population. The consumption of tobacco and alcohol has been widely recognized as a potential risk factor for oral cancer [5]. Oral cancer commonly results from potentially malignant lesions or normal epithelium linings. Potentially malignant disorders (PMDs) such as inflammatory oral submucosa, fibrosis, erythroplakia, leukoplakia, candidal leukoplakia, dyskeratosis congenital, and lichen planus are indicators of the preclinical phase of oral cancer. Thus, dental infections like periodontitis, periapical lesions, periapical granulomas, long-standing granulation tissues, and gingival swellings may present clinically as a common diagnostic finding [4]. Awareness about oral cancer, clinical findings, and the experience of the dental professional will usually form the basis of a successful diagnosis.

A delayed diagnosis leads to a subsequent treatment delay, which results in a poor prognosis of oral cancer in the long run [6,7]. The early diagnosis and proper treatment can improve the survival rate of the patient by up to 90% [7,8]. A wide array of procedures and techniques are available to assist in the diagnosis of various oral diseases. The clinical and radiographic examinations may provide sufficient information for the diagnosis of the clinical findings of common oral diseases. However, lesions of the oral cavity, especially those affecting the mucous membranes, may present unusual clinical patterns. This is essentially true because of the salivary environment, and the complex organization of the various oral membranes [7]. Thus, these clinical findings may be signs and symptoms of oral cancer that can only be confirmed through histological investigation. A lack of histological investigation often leads to delays in diagnosis or misdiagnosis of oral cancer.

A histopathological analysis is important to verify the proliferation of cells and maturation abnormalities, cellular and cytoplasmic atypia, and alteration of the surface epithelium or deep tissue cytoarchitecture. Proper identification and adequate sampling of the oral lesion is an important step in a histological investigation. Histopathological alterations can occasionally take place in regions where a physical examination reveals no signs of mouth lesions, and thus, the “gold standard” for diagnosing oral lesions has been established to be the histological examination of a tissue biopsy. Dental professionals largely rely on the histological examination of these incisional biopsies to help them make an accurate diagnosis and, consequently, plan the patient's treatment. In order to confirm the diagnosis of a suspected benign lesion, they also rely on the histological examination of excisional biopsies (e.g., fibroepithelial polyp, squamous papilloma) [7]. It is, thus, highly advised that all small tissue samples should be sent for histopathological confirmation and that a biopsy be required, particularly in patients with known risks for malignancy. 

Many dental researchers have missed dentists' liability for the issue as a result of the health approach's exclusive focus on patient responsibility for oral cancer, which is embodied in dentists' passivity towards oral cancer examination methods, patient education, and counselling [6]. Keeping all these considerations in mind, the current study's objective was to evaluate dentists' knowledge and attitudes regarding the diagnosis of oral cancer using histopathology of granulation tissue in Uttar Pradesh, India.

## Materials and methods

Study design and setting

This was a cross-sectional study conducted among 250 dentists practising or residing in Uttar Pradesh, India. The purpose of the questionnaire was to gather information on the prevalence, aetiology, incidence, and significance of biopsy and granulation tissue collection for further research to diagnose oral cancer. 

The study participants were selected based on a random sampling method. Dentists who gave their consent for the study and were willing to participate were included in the study, while those who did not want to participate were excluded from the study. The study participants were classified as academicians when they worked as full-time teaching faculty at a dental college or similar institution. The rest of them were classified as clinicians if they had their own private dental setup or worked in a clinical setting with no teaching experience in the field of dentistry. The intention of selecting academicians and clinicians for the study was to identify the knowledge gap and their accountability for the diagnosis and treatment of their patients.

The study was carried out over the course of three months, from June to August 2020. The Institutional Review Board of Divya Jyoti College of Dental Sciences and Research, Ghaziabad, India, approved the study (approval number: DJD/IEC/2020-A05). All study participants received a thorough explanation of the study's procedures, methods, and objectives before their informed consent was obtained.

Questionnaire

The study was carried out through a self-structured questionnaire comprising 15 closed-ended questions about the dentists' knowledge, attitudes, and practices regarding oral cancer. It was distributed among the study participants and they were given a week to complete the questionnaire. Demographic details including age, gender, educational attainment, years of clinical experience, and practice setting about the participants were collected. The responses were rated according to a three-point Likert scaleas: Agree = 2, Neither Agree nor Disagree = 1, and Disagree = 0.

To assess the viability of the present study and check the reliability of the questionnaire, a pilot study was carried out with 50 dentists who were interviewed face-to-face and were not involved in the main investigation. Cronbach's Alpha, a measure of internal consistency, showed a value of 0.87, which was regarded as good.

Statistical analysis

Microsoft Excel (Microsoft Corporation, Redmond, Washington, United States) was used to code and enter the results. IBM SPSS Statistics for Windows, Version 21.0 (Released 2012; IBM Corp., Armonk, New York, United States) was used to evaluate the data that had been collected. The results of the study participants' responses were compared using descriptive statistics and independent t-test. Independent t-test was used to compare the knowledge and attitude of the dental professionals of the two groups, Academician and Clinician. P-value is the probability that the alternative hypothesis is true. P≤0.05 was considered statistically significant.

## Results

The study included a sample size of 250 dentists; 21 (8.4%) dentists were below 25 years of age, 217 (86.8%) were aged 25-40 years, and the remaining 12 (4.8%) were over 40 years of age. The mean age of the study population was 31.564 ± 6.344 years. The majority of the respondents were males (60.8%). Most of the respondents (61.6%) had one to two years of experience as a dentist and 91.6% had a private dental practice. Most of the study subjects (68.8%) were dental practitioners as compared to 31.2% who were academicians (Table 1).

**Table 1:** Demographic factors of the study participants

Variable	Classification	N	Percentage
Age	Below 25 Years	21	8.4%
	25-40 Years	217	86.8%
	Above 40 Years	12	4.8%
Gender	Male	152	60.8%
	Female	98	39.2%
Year of Experience	Less Than 1 Year	54	21.6%
	1-5 Years	154	61.6%
	More Than 5 Years	42	16.8%
Type of Practice Setting	Government	18	7.2%
	Private	229	91.6%
	Voluntary Organization (Ngo)	3	1.2%
Type of Dentist	Clinician	172	68.8%
	Academician	78	31.2%

Most participants (94.4%) in the current study were aware of the fact that cancer is the second most common cause of death in India, and 95.6% of those surveyed were aware that cancer of the mouth is one of the most common cancers in India. The different premalignant lesions and diseases linked to oral cancer, as well as the potential risk factors for oral cancer, were known to 84.4% of study participants. Thirty-seven dentists (14.8%) dentists encountered at least one patient with a history of improper screening, incorrect diagnosis, or delayed detection of oral cancer, which was determined to be statistically significant between the two groups of dentists, practitioners and academicians (Table 2).

**Table 2:** Knowledge, attitude, and practice (KAP) of participating dentists for the diagnosis of oral cancer using histopathological investigation SD: standard deviation

S.No	Questions	Agree (2)	Neither Agree nor Disagree (1)	Disagree (0)	Mean ± SD
1	Do you realise that in India, cancer ranks as the second leading cause of death?	236 (94.4%)	3 (1.2%)	11 (4.4%)	1.90 ± 0.42
2	Do you know that one of India's top three cancers is oral cancer?	239 (95.6 %)	3 (1.2%)	8 (3.2 %)	1.92 ± 0.37
3	Are you aware of the potential risk factors of oral cancer?	242 (96.8%)	2 (0.8%)	6 (2.4%)	1.94 ± 0.32
4	Are you aware of the various premalignant lesions and conditions?	212 (84.8%)	4 (1.6%)	34 (13.6%)	1.71 ± 0.69
5	Have you ever encountered a patient with a history of negligence in screening, misdiagnosis, or delayed diagnosis of oral cancer by a dentist?	37 (14.8%)	2 (0.8%)	211 (84.4%)	0.30 ± 0.71
6	Do you think it is essential to take a biopsy to diagnose oral cancer?	245 (98.0%)	3 (1.2%)	2 (0.8%)	1.97 ± 0.21
7	Are you aware of the indications and contraindications of different biopsy techniques?	198 (79.2%)	3 (1.2%)	49 (19.6%)	1.59 ± 0.80
8	Do you know the various types of biopsy?	223 (89.2%)	2 (0.8%)	25 (10.0%)	1.79 ± 0.60
9	Do you know the importance of biopsy?	118 (47.2%)	2 (0.8%)	130 (52.0%)	0.95 ± 0.99
10	Have you ever done a biopsy and sent a tissue for histopathological examination?	36 (14.4%)	0 (0.0%)	214 (85.6%)	0.29 ± 0.70
11	Are you aware that oral cancer can appear in the form of periapical lesions, periapical granulomas, granulation tissues, gingival swellings, etc.?	25 (10.0%)	2 (0.8%)	223 (89.2%)	0.21 ± 0.60
12	Do you send granulation tissues associated with the tooth after extraction for further investigative procedures?	47 (18.8%)	0 (0%)	203 (81.2%)	0.38 ± 0.78
13	How many times has your provisional diagnosis been different from a biopsy diagnosis given by an oral pathologist?	163 (65.2%)	0 (0%)	87 (34.8%)	1.30 ± 0.95
14	Is the survival rate low in patients with advanced cancer, even after treatment with adjuvant modalities such as radiation therapy or chemotherapy?	213 (85.2%)	2 (0.8%)	35 (14.0%)	1.72 ± 0.69
15	Do you agree that early detection of oral cancer would not only improve cure rates but also reduce the costs and morbidity associated with treatment?	246 (98.4%)	0 (0%)	4 (1.6%)	1.97 ± 0.25

However, in the current study, only a few (47.2%) dental professionals knew the importance of biopsy to diagnose oral cancer and even fewer (14.4%) had done a biopsy and sent a tissue for histopathological examination. Of the study participants, 79.2% were aware of the indications and contraindications of different biopsy techniques, which was found to be statistically significant. Furthermore, only 18.8% of the study subjects had sent granulation tissue associated with extracted teeth for any histopathological investigation, which was found to be statistically significant for the two groups.

Merely 10.0% of dentists knew that malignancies could also arise from periapical granulomas, granulation tissues, gingival swellings, etc., which are frequently encountered in clinical practice. Of these dental professionals, 65.2% had observed that their provisional diagnosis related to oral cancer was different from a biopsy diagnosis given by an oral pathologist, which necessitates the histopathological study of these tissues. 

Most of the study participants (85.2%) believed that survival in patients with advanced cancer was poor, even after treatment with adjuvant modalities. Most of them (98.4%) agreed that early detection of oral cancer would not only help improve cure rates but would also reduce the cost and morbidity associated with oral cancer treatment, which was found to be statistically significant for the two groups (Table 3).

**Table 3:** Comparison of the mean knowledge, attitude, and practice (KAP) scores of the participating dentists between the two groups - Practitioners and Academicians * Significant (p≤0.05)

S.No	Questions	Mean Difference	T value	P value
1	Do you realise that in India, cancer ranks as the second leading cause of death?	-0.03	-0.48	0.330
2	Do you know that one of India's top three cancers is oral cancer?	-0.04	-0.74	0.135
3	Are you aware of the potential risk factors of oral cancer?	0.05	-0.72	0.148
4	Are you aware of the various premalignant lesions and conditions?	-0.09	-0.92	0.063
5	Have you ever encountered a patient with a history of negligence in screening, misdiagnosis, or delayed diagnosis of oral cancer by a dentist?	0.20	1.93	0.000*
6	Do you think it is essential to take a biopsy to diagnose oral cancer?	0.00	0.14	0.760
7	Are you aware of the indications and contraindications of different biopsy techniques?	-0.16	-1.43	0.003*
8	Do you know the various types of biopsy?	-0.07	-0.79	0.112
9	Do you know the importance of biopsy?	-0.06	-0.45	0.327
10	Have you ever done a biopsy and sent a tissue for histopathological examination?	-0.03	-0.32	0.524
11	Are you aware that oral cancer can appear in the form of periapical lesions, periapical granulomas, granulation tissues, gingival swellings, etc.?	-0.08	-0.88	0.092
12	Do you send granulation tissues associated with the tooth after extraction for further investigative procedures?	-0.12	-1.10	0.037*
13	How many times has your provisional diagnosis been different from a biopsy diagnosis given by an oral pathologist?	0.16	1.13	0.048*
14	Is the survival rate low in patients with advanced cancer, even after treatment with adjuvant modalities such as radiation therapy or chemotherapy?	0.07	0.74	0.137
15	Do you agree that early detection of oral cancer would not only improve cure rates but also reduce the costs and morbidity associated with treatment?	0.08	2.30	0.000*

## Discussion

Oral cancer has a significant negative impact on an individual's oral and general health, as is well documented in the literature. In 2018, there were reported to be around 18.1 million new instances of oral cancer worldwide, and there were 9.6 million fatalities [9]. In India, oral cancer is a significant cause of disease and mortality and is quite costly. An oral tissue biopsy is the current gold standard for identifying oral cancer [8]. The present study is distinctive because there are only a few similar studies in the literature that focus on the knowledge and attitude of dental professionals regarding the diagnosis of oral cancer through histopathological examination of granulation tissue.

In the current study, it was observed that 94.4% of the study subjects were aware that cancer is the second most common cause of death in India, and 95.6% were also aware that cancer of the mouth is one of the most common cancers in India. Surprisingly, only a few (47.2%) participants knew the importance of biopsy to diagnose oral cancer and even fewer (14.4%) had done a biopsy and sent a tissue for histopathological examination. The current study also showed that the majority (81.2%) of dental professionals had never sent granulation tissues for histopathological investigation.

These study findings were in agreement with the study conducted by Bataineh et al., which reported that 93.8% of the participants claimed to have the ability to diagnose oral mucosal lesions, 91.4% recognized the diagnostic importance of an oral biopsy, and 67.0% knew the indications of performing an oral biopsy, while only 30.7% considered oral biopsy as a diagnostic method to be used to reach a diagnosis of an oral mucosal lesion. These study findings strongly indicate that a significant difference exists between the perceived theoretical knowledge related to oral biopsy and the clinical practical application of this knowledge among the participant dentists [10].

In a study conducted by Tyagi et al., it was observed that when an oral mucosal abnormality was suspected in patients, 60.0% of practitioners routinely advised such patients for biopsy whereas 5.45% of dentists did not advise biopsy for their patients. Only 17.2% of the dentists reported that they performed biopsies on their patients themselves. It was shockingly revealed that 49.08% of the study subjects did not want to examine patients with mucosal lesions in their dental practice and out of these dentists, 44.44% did not even refer such patients to a specialist or to a higher speciality centre. Out of the study subjects who did examine and/or treat oral lesions, 82.14% used visual examination alone to arrive at a provisional diagnosis without any adjunctive aids used for arriving at the final diagnosis [11].

Cancer of the gingiva can occasionally develop after tooth extraction. However, upon careful examination of these cases, it could be typically possible to ascertain that the tooth was removed due to a tumour that was not recognised and also undiagnosed at the time of treatment, rather than a gum injury, disease, or mobility (surgery). Gingival cancer does not seem to have a more clear-cut or specific aetiology than any other cancer of the oral cavity. One could make assumptions about the potential role of chronic irritation in the development of gum cancer as the gums are the site of chronic irritation and inflammation [12]. In the current study, only 10% of the study subjects were aware of the fact that periapical granulomas, which are frequently encountered in clinical practice, could turn out to be actual malignancies in the oral cavity. Studies have reported that the granulation tissue left post extraction, which was thought to be a poorly healing or dry socket, turned out to be a malignant lesion. The sudden proliferation of cancerous tissue after tooth extraction and unrestricted growth of neoplastic tissue along the periodontium are likely to be blamed for the rapid development and spread of the carcinoma in some cases after tooth extraction [13].

In a study conducted by Murgod et al., it was reported that all the study subjects (100%) felt that biopsy was an important tool in the diagnosis of oral lesions, but many still did not venture to undertake it on their own [14]. The study reported that most of the study subjects either called a specialist for biopsy (34.33%) or referred them to a higher centre (31.34%). Only a small percentage (14.93%) said that they perform the biopsies themselves. This was mainly due to a lack of experience and patient factors.

In the current study, most of the study participants (85.2%) believed that survival in patients with advanced oral cancer was poor, even after treatment with adjuvant modalities; 98.4% also agreed that early detection of oral cancer would not only help improve cure rates but would also reduce the cost and morbidity associated with oral cancer treatment. Studies have shown that around 1% of newly discovered oral cancers are brought on by metastatic tumours [15]. The prognosis has been determined to typically be not good and oral metastases are uncommon. In the first year, most patients pass away, with a four-year survival rate of only 10%.

Oral cancer could begin slowly and spread undetected, leading to misdiagnoses like persistent gingivitis, periodontitis, or other dental abscesses. Without a thorough investigation and necessary investigations, such as biopsies and X-rays, dealing with these masquerades could result in needless tooth extractions. Malignant lesions may also have classic benign clinical characteristics. Therefore, in order to give patients the right care, all surgical specimens must be sent for histological analysis [14]. Early detection of cancerous lesions would facilitate effective treatment and improve the patient's long-term outlook, reducing the morbidity and mortality rates related to oral cancer.

The results of the study should be interpreted accordingly while taking the various limitations of the study into consideration. The present study evaluated a small number of dental professionals residing in Uttar Pradesh, India. Thus, the present study cannot be generalized to the entirety of the dental professionals of India. With this limitation in mind, a comparison with previous studies in the literature can be attempted. A well-designed, longitudinal study with an increased sample size is required to further evaluate dental professionals' knowledge and attitude regarding the diagnosis of oral cancer through histopathological examination of granulation tissue.

The present study pointed out the need for improvement in the training programme at the undergraduate level as biopsy is considered the gold standard for the diagnosis of many oral lesions and should be incorporated into daily dental practice. An increased awareness of the role of oral pathologists as consultants in the clinical practice also needs to be stressed enough for the prompt diagnosis of oral cancer cases.

## Conclusions

The current study contributes to a growing body of research that suggests dentists need educational interventions in the areas of oral cancer detection, screening, and prevention. Despite being in the best position to examine, diagnose, and treat oral mucosal lesions, dentists are not always confident in their abilities to do so. Because dental professionals are not well-informed regarding the proper identification, value, and necessity of biopsy procedures for diagnostic purposes. It is believed that providing ongoing dental education programmes would significantly improve the early detection and prevention of oral cancer. The best educational initiatives should centre on risk factor screening, behaviour modification counselling, physical oral cavity examinations, and a review of the requirements for specialist referral for a biopsy, final diagnosis, and treatment.
